# Bridging the Postpartum Cliff—First Year Outcomes of a Postpartum Transition to Primary Care Clinic

**DOI:** 10.1177/26884844251379414

**Published:** 2025-09-22

**Authors:** Radhika Malhotra, Aashka Parikh, Natalie Sous, Pauline Thomas, Lisa Gittens-Williams, Mirela Feurdean

**Affiliations:** ^1^ Department of Medicine, Rutgers New Jersey Medical School, Newark, New Jersey, USA.; ^2^ Department of Preventive Medicine, Rutgers New Jersey Medical School, Newark, New Jersey, USA.; ^3^ Department of Obstetrics, Gynecology, and Reproductive Health, Rutgers New Jersey Medical School, Newark, New Jersey, USA.

**Keywords:** postpartum cliff, primary care, postpartum transition, patient navigation

## Abstract

**Background:**

Half of maternal deaths occur after 42 days postpartum, a time when women are already out of obstetrical care. The American College of Obstetricians and Gynecologists recommends postpartum transition to primary care within 12 weeks of delivery. The majority of women do not transition to primary care, even those with chronic conditions like hypertension and diabetes. Those who do may experience the “postpartum cliff,” a drop-off in communication between obstetrician-gynecologist (OB/GYN) and primary care provider (PCP).

**Objective:**

The purpose of this study is to assess attendance rates at primary care appointments among high-risk postpartum patients who were referred through an enhanced postpartum referral system and to evaluate follow-up care in the early postpartum period.

**Methods:**

A dedicated “Healthy Moms Clinic” (HMC) and referral protocol were established using patient navigators between maternal fetal medicine (MFM) and primary care in January 2023. A retrospective chart review was conducted in November 2024 of women who were referred from MFM to primary care. The primary outcome variable was attendance rate at the HMC. Secondary outcomes included preventive screenings, contraception use, and management of chronic conditions such as hypertension and diabetes.

**Results:**

Of 106 referrals between January 2023 and July 2024, 93.4% attended their 6-week postpartum OB/GYN visit. Half (53.8%) attended the initial PCP visit, and 30.2% of the missed appointments were rescheduled. If the visit was rescheduled, half of those patients attended their rescheduled appointment with an overall show rate of 70.8%. There was no difference in show rates by race/ethnicity (patients identified primarily as Black or Hispanic) nor by insurance type.

**Conclusion:**

Coordination between obstetrics and primary care through dedicated transition clinics allows interdisciplinary collaboration, providing a solution for missed care postpartum. More time is needed to assess long-term outcomes such as hypertension control, diabetes control, and weight loss.

## Introduction

The United States is experiencing a maternal morbidity and mortality crisis, with rates far higher than other well-resourced countries. This disproportionately affects Black and Hispanic mothers, who are two to three times more likely to die during and following pregnancy than White women.^1,2^ Half of maternal deaths occur between 7 days and 1 year after delivery.^3,4^ Leading causes of maternal morbidity and mortality are cardiovascular disease, cardiomyopathy, mental health diseases, and postpartum hemorrhage.^2^ Women who have cardiovascular complications during pregnancy are at higher risk for long-term cardiovascular complications later in life and need continued monitoring.^5^ The same applies for diabetic disorders of pregnancy, which are associated with later development of type 2 diabetes.^6^ Based on these factors, the American College of Obstetricians and Gynecologists (ACOG) recommends comprehensive postpartum follow-up care and transition to primary care.^7^ Fewer than half of women in the United States used primary care doctors in 2018.^8^ Pregnancy is often the first time women are interacting with the health care system and is an opportune moment to screen for and engage women diagnosed with chronic conditions.^9^ Much less information is known about postpartum women following up in primary care. Around half of patients with hypertensive disorders of pregnancy (HDP) have been shown to follow up in primary care within 6 months postpartum; however, this number is much lower for Black and Hispanic women and women without commercial insurance.^10^ For persons with diabetes, follow-up rates with primary care postpartum were even lower at 10%–30%.^11,12^ Limited studies have shown that insurance access is one of the biggest predictors of transitions to care.^10,13^ Furthermore, postpartum follow-up occurs mainly when women have a preexisting relationship with a primary care physician.^10^ Women struggle with whom to call for their own care after they deliver, leaving confusion and putting the onus on the patient to determine their follow-up plan.^14^

The period after the 42nd day postpartum, a time when women normally attend their postpartum appointment at their obstetrician-gynecologist, is often described as the “postpartum cliff,” where there is lack of transition between obstetrical and primary care.^15^ Even when this transition is made, care is often fragmented and uncoordinated between services.^14^ Postpartum navigation is associated with increased postpartum care and decreased hospital readmission.^16^ We have previously briefly described an innovative transition of care referral system for high-risk maternal patients to primary care, a pilot program focused on improving postpartum engagement and continuity of care in a tertiary care center at University Hospital in Newark, NJ.^17^ Previous studies at our hospital site have shown that Black women with hypertension were less likely to attend their postpartum follow-up than White women, and that women with pregestational diabetes who achieved glycemic control during the pregnancy started a subsequent pregnancy with elevated hemoglobin A1C.^18,19^ This suggested a crucial need in our patient population to incorporate primary care follow-up into their postpartum care plan and help mitigate this fragmented access to care in between pregnancies, which led to the creation of the referral system. The purpose of this study is to assess attendance rates at primary care appointments among high-risk postpartum patients who were referred through an enhanced postpartum referral system and to evaluate follow-up care in the early postpartum period.

## Methods

We implemented a postpartum transitions of care referral system between the maternal fetal medicine (MFM) department and the primary care clinics (the internal medicine [IM] residency practice and the faculty practice medicine-pediatrics), establishing the Healthy Moms Clinic (HMC) at University Hospital in January 2023. The goal of the clinic was to ensure that women obtain optimal care beyond the recommended obstetrician-gynecologist (OB/GYN) postpartum visit for management of their chronic medical issues. Prior to this intervention, high-risk patients were instructed to follow up with primary care. However, there was no formal relationship arranged between the high-risk and primary care clinics at our institution, and primary care follow-up appointments were not scheduled prior to delivery.

**FIG. 1.**
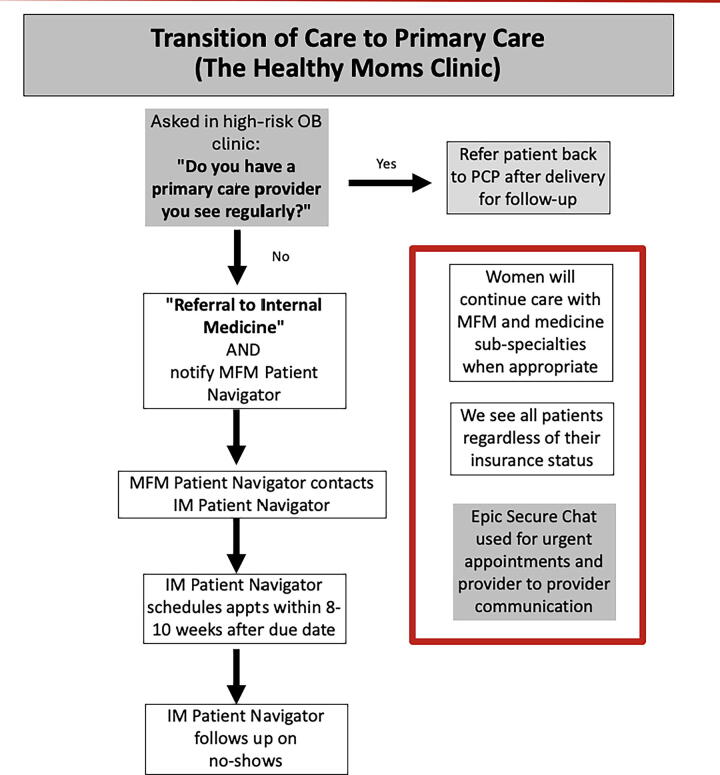
Workflow for postpartum transitions of care referral system created between maternal-fetal medicine and primary care providers at a clinic in University Hospital. PCP, primary care physician; MFM, maternal fetal medicine.

The referral process, described in Figure 1, highlights the change in existing practice patterns. High-risk patients seen in MFM were asked if they saw a primary care provider (PCP) regularly. If they did, the patients were counseled regarding the importance of primary care after delivery and referred to their existing PCP. If they did not have an existing PCP, they were referred to the HMC by the MFM patient navigator. The role of the MFM patient navigator was to help facilitate the referral and reinforce the importance of attending the appointment to the patient. The MFM patient navigator then coordinated the referral with the IM patient navigator. The IM patient navigator reached out to the patient to schedule the initial appointment per patient preference for mother-only or mother–baby appointment. This appointment was scheduled between 10 and 12 weeks postpartum to ensure adequate follow-up after the OB/GYN postpartum appointment at 8 weeks. Given that all our referrals were high-risk patients, all appointments were scheduled with this consistency. The IM medical technicians conducted reminder calls prior to the appointment. Epic secure chat was also used for urgent appointments and provider-to-provider communication when indicated. Importantly, women continued care with subspecialties when appropriate.

A “no-show” protocol was created as well. If the patient did not attend their primary care appointment, the patient reentered the workflow for the IM coordinator to reschedule the appointment for a later time. Periodic audits every quarter are additionally conducted by the program coordinator to ensure that referred patients are not lost and multiple attempts were made to reschedule appointments. After the third no-show, patients were no longer rescheduled in the clinic. A standardized note template was created for the clinic that addresses ACOG and United States Preventive Services Task Force recommendations for primary care visits. Depression and domestic violence screenings were performed at the primary care visit. Gynecological care, such as birth spacing, breastfeeding, and contraception, was readdressed at the primary care visit. Risk factors of cardiovascular disease were addressed with management of hypertension and diabetes, hyperlipidemia screenings, and weight management discussions. Routine cancer screenings and vaccinations were also addressed.

A retrospective chart review was conducted using electronic health record data of women who were referred to the HMC between January 2023 and July 2024. The retrospective chart review was conducted in November 2024. Women who were referred from MFM to the HMC were included in this study. Any women outside of the referral system and those with existing PCPs outside of University Hospital were excluded from the study. The primary outcome of interest was show rates at appointments, both at the postpartum OB/GYN visit and the primary care visit. This included show rates after implementing the no-show policy. Baseline characteristics such as age, median income by zip code of residence, number of prenatal visits, race, ethnicity, insurance type, and primary language were collected. Data regarding their pregnancy-related chronic conditions (*e.g.,* hypertension and diabetes), screenings performed at the PCP visit, contraception use, and medication adjustments performed at the OB/GYN visit and primary care visit were analyzed.

Patients who attended the primary care visit were compared with those who did not attend the primary care visit after attempts at rescheduling. Demographic factors, such as race, ethnicity, insurance type, and primary language, were compared, as well as presence of the two most common chronic conditions, hypertension and diabetes. Hypertension characteristics, such as if the patient was on antihypertensives during pregnancy or re-admitted for hypertension postpartum, were also compared with assessments for severity of disease. Blood pressure control at the primary care visit was defined by systolic blood pressure (SBP) <120 mmHg and diastolic blood pressure (DBP) <80 mmHg based on recommendations by the American College of Cardiology and American Heart Association.^20^ Diabetes type and control were also analyzed for similar reasons. For associations between categorical variables, either student’s independent *t*-tests or analysis of variance (ANOVA) was done. A two-sided *p*-value of <0.05 was used for statistical significance. Data analysis was conducted using IBM Statistical Package for the Social Sciences version 27.0 for Mac. This study was approved by the Rutgers University Institutional Review Board.

## Results

### Characteristics of high-risk women referred from maternal-fetal medicine clinic

A total of 106 women were referred from MFM to the HMC, 84 of whom were referred to IM and 22 were referred to medicine-pediatrics. Demographics and other characteristics of the women referred are shown in Table 1. The average age of patients referred was 33.5 years old. This was a primarily Black non-Hispanic (*n* = 40, 37.7%) and Hispanic/Latino (*n* = 55, 51.9%) population. Most patients were Medicaid recipients (*n* = 75, 70.8%,) uninsured, or uninsured and recipients of NJ Charity Care (27.4%). Primary language spoken was mostly English (*n* = 42, 39.6%) or Spanish (*n* = 49, 46.2%).

**Table 1.** Demographics of 106 Patients Who Were Referred to the Healthy Moms Clinic

Clinic type	No. (%)
Clinic patients were seen in	
IM clinic	84 (79.2%)
Med/peds clinic	22 (20.8%)
** **	Average ± standard dev
Demographics	
Age	33.5 ± 5.7
Median income	51,472 ± 9,834
BMI at beginning of pregnancy	32.6 ± 8.91
BMI at end of pregnancy	34.6 ± 8.3
Gravidity (average)	3.4 ± 2.8
Parity (average)	3.0 ± 1.6
** **	No. (%)
Race/ethnicity	
African-American or Black non-Hispanic	40 (37.7%)
Hispanic/Latino	55 (51.9%)
Other	11 (10.4%)
Insurance type	
Medicare/medicaid	75 (70.8%)
Private insurance	2 (1.9%)
None/self-pay/charity care	29 (27.4%)
Primary language as listed in chart	
English	42 (39.6%)
Spanish	49 (46.2%)
French	2 (1.9%)
Portuguese	1 (0.9%)
Haitian Creole	9 (8.5%)
French Creole	1 (0.9%)
Other	2 (1.9%)
Preexisting reported medical conditions	
None	21 (19.8%)
History of preeclampsia in a previous pregnancy	8 (7.5%)
History of GDM	1 (0.9%)
Asthma/COPD	9 (8.5%)
Depression/anxiety	2 (1.9%)
DM	17 (16.0%)
HTN	31 (29.2%)
Hypothyroidism	7 (6.6%)
Seizures	4 (3.8%)
Anemia	26 (24.5%)
Other	42 (39.6%)
Unknown	1 (0.9%)

BMI, body mass index; DM, diabetes mellitus; GDM, gestational diabetes mellitus; IM, internal medicine.

Table 2 shows information about the chronic conditions identified prior to pregnancy in the women. Thirty women (28.3%) had a diagnosis of chronic hypertension prior to being pregnant, with 16 of these (53.3%) on medications for chronic hypertension during the pregnancy. An additional 30 women developed a HDP. A total of 33 women (31.1%) had some form of preeclampsia. Three women had pregestational type 1 diabetes (7.0%), and 15 women (34.9%) had pregestational type 2 diabetes. An additional 53.5% (*n* = 23) had developed gestational diabetes. A small percentage had abnormal glucose tolerance tests (GCTs) during the pregnancy without a diagnosis of diabetes (*n* = 2, 4.6%).

**Table 2.** Hypertension and Diabetes Diagnoses for Women Who Were Referred to the Healthy Moms Clinic

Chronic hypertension	
Yes	30 (28.3%)
Hypertensive disorder of pregnancy and what type	
None	45 (42.5%)
Chronic hypertension	12 (11.3%)
Gestational hypertension	17 (16.0%)
Preeclampsia	4 (3.8%)
Severe preeclampsia	11 (10.4%)
Super-imposed preeclampsia	17 (16.0%)
On meds for chronic hypertension	
Yes	16 (15.1%)
Diabetes in pregnancy (*n* = 43)	
Gestational DM	23 (53.5%)
Pregestational Type 1 DM	3 (7.0%)
Pregestational Type 2 DM	15 (34.9%)
Abnormal GCT without diagnosis of DM	2 (4.7%)

GCT, glucose tolerance test.

Table 3 shows attendance rates at the HMC for the women who were referred. Of 106 women, 99 (93.4%) attended their postpartum visit with the obstetrician-gynecologist. Comparatively, only 54 women (50.9%) attended their initial scheduled primary care visit, either in the IM resident clinic or the medicine-pediatrics clinic. Of the 49 women who missed their original appointment, 61.5% (*n* = 32) had their visit rescheduled by the patient navigator, and of these, 46.9% (*n* = 15) attended their rescheduled visit. Fourteen women had previously received care with the resident IM primary care clinic and were referred back for timely postpartum care. 44.0% (*n* = 34) of women attended their follow-up visit as scheduled in the clinic within the study period. The total attendance rate for the clinic was 70.8% (*n* = 75).

**Table 3.** Attendance Rates at OB/GYN and Primary Care Appointments for the Patients Who Were Referred to the Healthy Moms Clinic

Postpartum visit at OB/GYN	
Attended?	99 (93.4%)
Total primary care attendance rate	
Yes, total show rate for first appointment	75 (70.8%)
Did patient attend original scheduled PCP visit?	
Yes	54 (50.9%)
If no showed, did reschedule?	
Was not rescheduled	22 (42.3%)
Rescheduled	32 (61.5%)
If visit rescheduled, did patient attend?	
Attended rescheduled visit	15 (46.9%)
Visited our primary care clinic prior to pregnancy?	
Yes	14 (13.2%)
Attend follow-up visit	
Yes (of 75 who attended their primary care visit)	33 (44.0%)

OB/GYN, obstetrician-gynecologist; PCP, primary care provider.

### Factors associated with adherence to healthy moms clinic primary care visits

A comparison of patients who did and did not attend their primary care appointment is shown in Table 4. There was no significant difference between race (ANOVA *p* = 0.93) or ethnicity (*t-*test *p* = 0.55). Rates of insurance were similar: among attendees, Medicaid *n* = 51, 68.0%, none/charity care *n* = 22, 29.3% versus among those who did not attend: Medicaid *n* = 24, 77.4%, none/charity care *n* = 7, 22.6%, ANOVA *p* = 0.4. Primary language was also similar between the groups, with equivalent numbers of English and Spanish speakers in both groups (ANOVA *p* = 0.27). There was no significant difference in attendance between women with chronic hypertension (*t-*test *p* = 0.45), women with HDP (ANOVA *p* = 0.10), or women on medications for chronic hypertension either before or during the pregnancy. There was also no difference in attendance rates between women who were readmitted for hypertension postpartum (ANOVA *p* = 0.54) or those who presented to the emergency department after delivery for hypertension (ANOVA *p* = 0.51). Having pregestational or gestational diabetes did not indicate likelihood to attend primary care visit (ANOVA *p* = 0.58). Most importantly, attending the postpartum OB/GYN visit was not an indicator of likelihood to attend the primary care visit in the HMC (*t-*test *p* = 0.3).

**Table 4.** Comparison of Demographics of Patients Who Attended the Primary Care versus Those Who Did Not

	Did not attend PCP appt	Attended PCP appt	*p-*Value
Race			
African-American or Black Hispanic	2 (6.5%)	1 (1.3%)	0.93
African-American or Black non-Hispanic	10 (32.3%)	30 (40.0%)	
Other	19 (61.3%)	44 (58.7%)	
Ethnicity			
Non-Hispanic or Latino	16 (51.6%)	38 (50.7%)	0.55
Hispanic or Latino	15 (48.4%)	37 (49.3%)	
Insurance Type			
Medicare/Medicaid	24 (77.4%)	51 (68.0%)	0.40
Private insurance	0 (0.0%)	2 (2.7%)	
None or self-pay or charity care	7 (22.6%)	22 (29.3%)	
Primary language			
English	12 (38.7%)	30 (40.0%)	0.27
Spanish	13 (41.9%)	36 (48.0%)	
French	0 (0.0%)	2 (2.7%)	
Portuguese	0 (0.0%)	1 (1.3%)	
Haitian Creole	5 (16.1%)	4 (5.3%)	
French Creole	0 (0.0%)	1 (1.3%)	
Other	1 (3.2%)	1 (1.3%)	
Chronic hypertension			
Yes	23 (74.2%)	53 (70.7%)	0.45
Hypertensive disorder of pregnancy			
Chronic hypertension	3 (9.7%)	9 (12.0%)	0.10
Gestational hypertension	3 (9.7%)	14 (18.7%)	
Preeclampsia	1 (3.2%)	3 (4.0%)	
Severe preeclampsia	2 (6.5%)	9 (12.0%)	
Super-imposed preeclampsia	4 (12.9%)	13 (17.3%)	
Medication for chronic hypertension			
Yes	7 (22.6%)	9 (12.0%)	0.14
Antihypertensives during pregnancy			
Yes	5 (16.1%)	12 (16.0%)	0.60
Readmitted for hypertension postpartum			
Yes	1 (3.2%)	4 (5.3%)	0.54
Presented to the ED within weeks after delivery for hypertension			
Yes	3 (9.7%)	9 (12.0%)	0.51
Gestational diabetes or pregestational diabetes?			
Gestational diabetes	8 (25.8%)	15 (20.0%)	0.69
Type 1 diabetes mellitus	0 (0.0%)	3 (4.0%)	0.047
Type 2 diabetes mellitus	5 (16.1%)	10 (13.3%)	0.95
Abnormal GCT	1 (3.2%)	1 (1.3%)	0.99
Was the diabetes well-controlled during the pregnancy?			
Not well-controlled	4 (30.8%)	16 (53.3%)	0.15
Did the patient attend the postpartum visit?			
Yes	28 (90.3%)	71 (94.7%)	0.33

### Care provided during first year of healthy moms clinic

We analyzed frequency of depression screenings that were performed in the postpartum period at primary care visits, the average number being 2 screenings. These screenings were either Edinburgh Postpartum Depression Scales or Patient Health Questionnaire (PHQ). Four women had positive depression screens at their primary care visit, indicating a need for further postpartum depression screening. Four additional women were continued on their long-term antidepressants at the primary care visit. Nine women had excessive weight gain during pregnancy (more than 25 pounds if normal BMI and more than 15 pounds if obese BMI) and were referred to the weight management center for optimal control (8.5%). 61.3% of the performed hyperlipidemia screenings (31 patients with screenings) had abnormal LDL levels (LDL >100) (*n* = 19). Most PCP visits had documented contraception counseling (*n* = 64, 87.7%), with only 14.2% of women not being on contraception after delivery (*n* = 15). Birth spacing was recommended at 100% of visits.

Table 5 provides information on medications and control of hypertension and diabetes both during and after the pregnancy. 27.9% of women (*n* = 17) were on antihypertensives during the pregnancy, and 21 patients had medications added at discharge by OB/GYN after delivery (34.4%). Among the women with HDP, many attended their scheduled primary care visit (*n* = 41, 67.2%), and of these, 53% had blood pressures that were uncontrolled (SBP >130 and DBP >90) at these visits. Medication adjustments were made at 50.8% (*n* = 31) of primary care visits, with medications being added (*n* = 9, 24.3%), changed (*n* = 10, 32.3%), or discontinued (*n* = 3, 9.7%).

**Table 5.** Medication Adjustments Made in the Primary Care Clinic for Patients with Hypertensive Disorders of Pregnancy and Diabetes Disorders of Pregnancy

Hypertension (*n* = 61)	
On hypertensive medication during pregnancy	17 (27.9%)
Hypertensive medication added at discharge after delivery	21 (34.4%)
Did patient attend scheduled primary care visit?	41 (67.2%)
Blood pressures at primary care visits well controlled?	19 (46.3%)
Medication adjustment at the primary care visit?	31 (50.8%)
Additional antihypertensive medication added?	9 (24.3%)
Diabetes (*n* = 43)	
Two-hour GCT collected at OB/GYN postpartum visit	13 (30.2%)
If completed, results abnormal?	7 (53.8%)
Attend the primary care visit?	23 (53.5%)
Continued on diabetic medications?	13 (30.2%)
	Average ± standard dev (*n*)
A1C postpartum	
Gestational diabetes	6.0 ± 0.5 (10)
Type 1 diabetes mellitus	6.5 ± 0.7 (3)
Type 2 diabetes mellitus	7.6 ± 1.9 (9)

There were 43 patients with a diabetes disorder of pregnancy, of which 17 were known to have type 2 diabetes prior to pregnancy. Nine were managed with oral agents (20.9%) and 20 with insulin (46.5%) during their pregnancy, while the remaining were diet controlled. 41.9% of patients were not well-controlled at their antepartum visits. Only 30.2% of diabetic women completed their postpartum 2-hour GCT at their OB/GYN visit, as seen in Table 5 (*n* = 13). Half of those with diabetes (*n* = 23, 53.5%) attended their primary care visit, and of these, 30.2% were continued on diabetic medications by their PCP. The average A1C following initial PCP visits was 7.6 in patients with known type 2 diabetes and 5.9 in patients with gestational diabetes. A fifth (21.8%) of patients needed additional diabetes medications postpartum to help control chronic diabetes.

## Discussion

We describe a referral system created between the high-risk maternal-fetal medicine clinic and the primary care IM clinic in an underserved, primarily Black and Hispanic community that is at disproportionate risk for maternal morbidity and mortality.^2^ We had a significant show rate of 70%, with a large percentage having hypertension or diabetes and clear need for ongoing treatment management. Unfortunately, we were not able to determine the rates of postpartum primary care follow-up prior to this intervention. We saw that around half of patients with hypertension had uncontrolled hypertension and that patients with type 2 diabetes mellitus (T2DM) had elevated A1Cs at their primary care visit.

Patients either had Medicaid or were uninsured/charity care patients and primarily either English or Spanish-speaking. These high-risk patients had overwhelmingly attended their postpartum OB/GYN appointment. This is higher than reported rates of postpartum OB/GYN attendance rates of 67% in 2021 in our patient population, showing that postpartum navigation helps increase immediate follow-up rate.^18^ In addition, our primary care postpartum follow-up rates are higher than national data in underserved populations such as ours.^21^ We found that around 70% of women who were referred attended the in-person primary care appointment, much higher than previously described postpartum primary care follow-up rates for minority women.^10,22^ The setting of our clinic is similar: minority women in similar inner-city settings. With the initiation of the referral system, we see an increased appointment completion rate. Similar rates have been described using telehealth as a model.^23^ Only a small percentage of these women had been to our IM clinic before, showing that the clinic was successful in new linkages to primary care. This interdisciplinary model based on electronic medical record prompt-based close loop communication allowed us to achieve successful transition to primary care. Outreach efforts for those who did not present to the initial appointment by a dedicated patient navigator to help them reschedule further improved follow-up rates. There was a small percentage who were not rescheduled, and we do not have data specifically on why that may be—it could be a barrier issue or a clinic-specific issue. Large-scale studies have previously shown that women with preexisting medical conditions struggle to make it to their 8-week follow-up postpartum visits.^24^ Using patient navigators to help facilitate the referrals was essential to helping our patients navigate the care system and utilize the resources available to them, as recommended in previous settings.^25,26^

Lack of insurance is a major barrier to follow-up care.^10^ Our patients were primarily either Medicare or uninsured or on charity care. Charity care in New Jersey implies financial need and needs to be renewed every year. Medicaid status or lack of health insurance has previously been identified as the strongest predictor for missing postpartum follow-up appointments.^27^ Despite their insurance status, we were able to engage women in postpartum follow-up and even return for their follow-up appointments, substantiating the benefits of navigation assistance. There was no difference in race, ethnicity, insurance type, primary language, or chronic condition between women who attended their postpartum appointment compared with those who did not. Attending the OB/GYN postpartum appointment was not an indicator of continued primary care.^10,12^ We believe that the barriers included lack of transportation, childcare issues, and system inefficiencies with decreased access to care.^28^ It is also very likely that the postpartum navigation process could further improve with increased patient education regarding the importance of primary care after a high-risk pregnancy. A next step will be to study these barriers by surveying our patient population and assessing their needs to improve our navigation process.

The high-risk population was primarily made up of women with HDP or pregnancy-related diabetes. Hypertension has been shown to be persistent at the 6-week OB/GYN visit in 20% of HDPs.^29^ Women with HDP are eight times as likely to have increased risk of chronic hypertension later in life, a risk that should be explained and discussed during pregnancy to help with navigation to primary care in the postpartum period.^30^ Medication adjustments were indicated in around 65% of hypertensive primary care visits and 60% of diabetic visits. This was in addition to changes that were made by the obstetrician at the postpartum OB/GYN visit. Physician titration of medications is associated with lower blood pressure in the first 9 months postpartum, supporting the value of primary care medication assistance in high-risk patients.^31^ Women with HDP are more likely to have increased cardiovascular risk factors 1 year after delivery.^32^ We found dyslipidemia in a large percentage of the population; it is frequently found in women with HDP and requires longer-term follow-up beyond the initial postpartum period.^33^ Linking women to primary care creates the opportunity for appropriate assessment of cardiovascular risk factors and behavioral counseling in the postpartum period and beyond.

Persons with diabetes disorders of pregnancy have similar progression of disease, as 70% of patients with gestational diabetes mellitus progress to T2DM within 5 years postpartum.^34^ As evidenced by our data and others, postpartum glucose tolerance testing with the OB/GYN has low completion rates.^35,36^ Even if patients complete their postpartum screening, follow-up annual screenings are decreased in subsequent years.^37^ However, it is important to mention that patients also struggle to complete laboratory testing due to lack of insurance, which can be a barrier to completing hemoglobin A1C screenings. More time and data are necessary and indicated to ascertain if our intervention provides improvement in diabetes control and outcome. However, we do overcome the inability of patients to be able to follow up in primary care by creating an avenue for transition.^38^

Depression screenings are recommended for all postpartum women, whether it be the Edinburgh or the PHQ scale.^39^ However, there is limited data regarding the screening rates for depression in primary care. In our clinic, we performed a depression scale at every visit. We found that four women had positive postpartum depression scores. These women were then referred to a social worker and looped into mental health resources to address possible social determinants of health and provide resources for support. As primary care providers, we are also able to manage the patient’s antidepressants and provide medical attention quickly.

The primary strength of this study and referral system lies in the fact that it can be easily reproduced at any clinic system across the country at relatively low cost. We were able to implement this referral change in an existing residency practice with existing staff. We demonstrate higher rates of follow-up care in our clinic than previous studies, which is likely a strength of the personalized referral system that directly links patient to care and creates appointments for them, removing undue burden. The key to establishing a successful transition of care lies in strong interdisciplinary teamwork and the ability to support patient navigation.

## Limitations

This study is a single-center study occurring at one site, and although the study is largely generalizable due to its feasibility and ease of implementation, we cannot generalize the results on a larger scale, particularly in a nonacademic setting. Importantly, we did not conduct pre-intervention data collection and therefore cannot draw conclusions about the effectiveness of this intervention in changing practice patterns. Furthermore, the HMC had been in operation for about 18 months, too soon to make conclusions regarding the improvement of overall health outcomes of our patient population, and we need several more years of data to specifically discuss outcomes related to hypertension and weight loss. We are not able to analyze the rates of postpartum referral and follow-up of women who had existing primary care physicians and were not referred to our clinic. Furthermore, we do not have data from the 30% of women who missed their appointments, and further qualitative research should be done to determine what barriers to care they may have faced. Further studies should measure how many women had accessed primary care prior to their high-risk pregnancy to evaluate the gap in care and if optimal preconception counseling had occurred.

## Conclusion

In conclusion, we have found that implementation of a postpartum transitions of care referral system can be achieved through interdisciplinary teamwork between obstetricians and primary care internists without much strain on an existing health system. Through navigation and a referral system, we created access to primary care in a particularly underserved and primarily Black and Hispanic population without prior established primary care and created a bridge to overcome the “postpartum cliff” by carrying women from obstetrical to primary care. The referral process linked women without prior primary care and created opportunity for continued care of their chronic conditions. More time is needed to evaluate the success of our clinic in long-term health outcomes, including hypertension control, diabetes control, weight loss, and mental health, all factors that can help increase cardiovascular mortality later in life.

## Abbreviations Used

A1C: Hemoglobin A1C (glycated hemoglobin test)
ACOG: American College of Obstetricians and Gynecologists
ANOVA: Analysis of Variance
BMI: Body Mass Index
COPD: Chronic Obstructive Pulmonary Disease
DBP: Diastolic Blood Pressure
DM: Diabetes Mellitus
ED: Emergency Department
GCT: Glucose Tolerance Test
GDM: Gestational Diabetes Mellitus
HDP: Hypertensive Disorders of Pregnancy
HHS: U.S. Department of Health and Human Services
HMC: Healthy Moms Clinic
HRSA: Health Resources and Services Administration
HTN: Hypertension
IM: Internal Medicine
LDL: Low-Density Lipoprotein
MFM: Maternal Fetal Medicine
NJ: New Jersey
OB/GYN: Obstetrician-Gynecologist
PCP: Primary Care Provider
PHQ: Patient Health Questionnaire
PRAMS: Pregnancy Risk Assessment Monitoring System
SBP: Systolic Blood Pressure
T2DM: Type 2 Diabetes Mellitus
USPSTF: U.S. Preventive Services Task Force
